# De Novo Transcriptome Identifies Olfactory Genes in *Diachasmimorpha longicaudata* (Ashmead)

**DOI:** 10.3390/genes11020144

**Published:** 2020-01-29

**Authors:** Liangde Tang, Jimin Liu, Lihui Liu, Yonghao Yu, Haiyan Zhao, Wen Lu

**Affiliations:** 1 Key Laboratory of Integrated Pest Management on Tropical Crops, Ministry of Agriculture and Rural Affairs, Environment and Plant Protection Institute, Chinese Academy of Tropical Agricultural Sciences, Haikou 571101, China; tangldcatas@163.com; 2 Guangxi Key Laboratory for Biology of Crop Diseases and Insect Pests, Institute of Plant Protection, Guangxi Academy of Agricultural Sciences, Nanning 530007, China; ljimin@126.com (J.L.); yangmeiliu@yeah.net (L.L.); yxp1127@163.com (Y.Y.); 3 Department of Entomology, College of Tobacco Science, Guizhou University, Guiyang 550025, China; 4 Guangxi Academy of Agricultural Sciences, Nanning 530007, China; 5 College of Agriculture, Guangxi University, Nanning 530007, China

**Keywords:** *Diachasmimorpha longicaudata*, Ashmead, parasitoid wasps, transcriptome, olfactory protein, odorant-binding protein, chemosensory protein

## Abstract

*Diachasmimoorpha longicaudata* (Ashmead, *D. longicaudata*) (Hymenoptera: Braconidae) is a solitary species of parasitoid wasp and widely used in integrated pest management (IPM) programs as a biological control agent in order to suppress tephritid fruit flies of economic importance. Although many studies have investigated the behaviors in the detection of their hosts, little is known of the molecular information of their chemosensory system. We assembled the first transcriptome of *D. longgicaudata* using transcriptome sequencing and identified 162,621 unigenes for the Ashmead insects in response to fruit flies fed with different fruits (guava, mango, and carambola). We annotated these transcripts on both the gene and protein levels by aligning them to databases (e.g., NR, NT, KEGG, GO, PFAM, UniProt/SwissProt) and prediction software (e.g., SignalP, RNAMMER, TMHMM Sever). CPC2 and MIREAP were used to predict the potential noncoding RNAs and microRNAs, respectively. Based on these annotations, we found 43, 69, 60, 689, 26 and 14 transcripts encoding odorant-binding protein (OBP), chemosensory proteins (CSPs), gustatory receptor (GR), odorant receptor (OR), odorant ionotropic receptor (IR), and sensory neuron membrane protein (SNMP), respectively. Sequence analysis identified the conserved six Cys in OBP sequences and phylogenetic analysis further supported the identification of OBPs and CSPs. Furthermore, 9 OBPs, 13 CSPs, 3 GRs, 4IRs, 25 ORs, and 4 SNMPs were differentially expressed in the insects in response to fruit flies with different scents. These results support that the olfactory genes of the parasitoid wasps were specifically expressed in response to their hosts with different scents. Our findings improve our understanding of the behaviors of insects in the detection of their hosts on the molecular level. More importantly, it provides a valuable resource for *D. longicaudata* research and will benefit the IPM programs and other researchers in this filed.

## 1. Introduction

*Diachasmimoorpha longicaudata* (Ashmead, *D. longicaudata*) is a solitary species of parasitoid wasp of several fruit fly species and has been introduced to many countries as a biological control agent. Its host, *Bactrocera dorsalis* Hendel, can attack many fruit species and some other plants, such as Caricaceae, Moraceae, Myrtaceae, Rosaceae, and Solaneaceae [1]. It is said that the female Ashmead insects can detect the fly larvae by sound in rotting fruit and that the attractant could be the fungal fermentation products rather than the chemical substances produced by the fly larvae [2,3]. Carrasco and colleagues reported that the presence of fly larvae was essential for the orientation of wasps [4]. Further, it was proposed that cues from the fruit can be used by the wasps directly and that the presence of the host can enhance the attraction towards a patch [5]. Interestingly, chemical compounds produced by the larvae can be detected by wasps to locate the host [6,7]. Once the female parasitoid is on the fruit, a specific chemical compound released by some Tephritidae species can be used to enhance the host search [7]. However, much is unknown about the chemosensory system of parasitoid wasps in response to their hosts. 

Animals can use the chemosensory system to detect and discriminate chemical cues in the environment [8]. The chemical sensors of insects are mainly from the antennae system, which is a highly specific and extremely sensitive chemical detector, and olfactory proteins in the antennae can be used by the insects to detect very low-abundance odorants from thousands of odors and further to guide their behaviors, such as forage, mate hunting, host plant location, shelter, and selection of spawning sites [9]. Since the first odorant-binding protein (OBP) was identified in *Antheraea polyphemus* [10], research on insect OBP has become a hot spot in the field of entomology. However, OBP-related studies have mainly been demonstrated in some important pests [11], and very few have been reported in parasitoid wasps, which are very important natural enemies of the pests.

The chemoreception of insects involves three important events: (i) The uptake of signal molecules from the external environment; (ii) transport (diffusion) through the sensory hair; and (iii) interaction with the chemoreceptor, which in turn activates the cascade of events leading to spike activity in sensory neurons [12]. Some important protein families have been reported to participate in these events, such as OBP, sensory neuron membrane protein (SNMP), chemosensory protein (CSP), odorant receptor (OR), gustatory receptor (GR), and odorant ionotropic receptor (IR) [12]. Insect OBPs are small globular proteins (~135 to 220 amino acids) and are characterized by a specific domain that comprises six α-helices joined by three disulphide bonds [12]. They can be categorized into two subgroups: Pheromone-binding proteins, which are mainly distributed in the male antenna, and general OBP (GOBP), which can be found in multiple tissues of male and female insects and function in the recognition of odorants from plants and other animals [13]. There are about 300 OBPs in the NCBI database and not many studies have been demonstrated to identify the OBPs in parasitoid wasps. Xu and colleagues used transcriptome sequencing and identified 1 CSP, 21 OBPs, 53 ORs, 29 IRs, and 4 SNMPs in *Bactrocers minax* [14], an oligophagous tephritid insect whose host selection, and oviposition behavior largely depend on the perception of chemical cues. Zhu et al. reported Sgua-OBP1 and Sgua-OBP2 in *Scleroderma guani* (Hymenoptera: Bethylidae) and NvitOBP in *Nasonia vitripennis* [15]. Zhang identified 10 OBPs in *Microplitis mediator* (Halidag) [16]. Zhao et al. identified 25 OBPs, 80 ORs, 10 IRs, 11 CSP, 1 SNMPs, and 17 GRs in adult male and female *Chouioia cunea* antennae [17]. However, little is known about the olfactory proteins in *D. longicaudata*.

Harbi and colleagues demonstrated a multistep assay (e.g., olfactory, laboratory, and semi-field trials) and reported the preference of medfly-infected fruits, including apple, orange, peach, and clementine mandarins [18]. This experiment supports that different olfactory genes are expressed in response to different fruit scents. In this study, we used transcriptome sequencing to study the olfactory genes in *D. longicaudata*. By similarity, we identified a number of OBPs, CSPs, ORs, IRs, SNMPs, and GRs expressed in the Ashmead insects. Our results also showed that different olfactory genes were expressed in the search of their host with different fruit scents. This is the first time to identify the gene and protein sequences for the olfactory products in this species. Our findings will provide a basis for future molecular studies and improve our understanding of the chemosensory system of parasitoid wasps.

## 2. Materials and Methods

### 2.1. Insect Rearing

The Ashmead and fruit fly larvae were obtained from the Institute of Plant Protection (IPP), Hainan Academy of Agricultural Sciences and maintained in the experimental fields. The fruit flies were fed with guava (G), mango (M), and carambola (C) separately. A mixture of yeast and sucrose (1:1) was used as supplementary nutrition for the fruit flies in the adult stage. Then, late-second and early-third instar fruit flies were used as hosts for the Ashmead insects. The Ashmead insects were fed with 15% honey water and clean water; the fifth, sixth and seventh generations of the Ashmead adults, which were parasitic to the fruit flies, maintained with G (G1~G3), M (M1~M3), and C (C1~C3), were used as biological replicates. The antenna, head, breast, abdomen, and feet tissues of the one male and two female wasps were mixed together for RNA extraction.

### 2.2. RNA Isolation, Library Construction, and Deep Sequencing

Total RNA was extracted from the insect tissues using the TRIzol reagent, as previously described [19,20]. The quality and quantity of total RNA were determined by multiple instruments, including a Nanodrop 2000 (Thermo Scientific, MA, USA), Qubit 4 Fluorometer (Invitrogen, CA, USA), and Agilent 2100 Bioanalyzer. Then, the total RNA (1 μg) of each sample was used to build the cDNA library using the TruSeq RNA Library Preparation Kit v2 protocol (Illumina, CA, USA), as described [20]. After the cDNA libraries were quality controlled by the Agilent 2100 Bioanalyzer and qRT-PCR, they were sequenced on the Illumina HiSeqXTEN platform with the paired-end 150 strategy. Raw sequencing reads of these samples can be accessed from the NCBI SRA platform under the accession numbers SRR10766480~SRR10766488.

### 2.3. Transcriptome Assembly

Raw sequencing reads were cleaned using the trim_galore v0.5.0 and the clean data was quality controlled by FASTQC v0.11.7 (http://www.bioinformatics.babraham.ac.uk/projects/fastqc/). Then, clean reads were used to assemble the transcriptome for each sample using Trinity software with default parameters, as described [21]. In detail, high quality RNA-Seq reads were used to generate overlapping k-mers (25) and Inchworm was used to assemble sorted k-mers into transcript contigs based on the (k-1)-mer overlaps. Next, Chrysalis was used to cluster related Inchworm contigs into components by using grouped raw reads and paired read links. Then, a de Bruijn graph for each cluster was built by Chrysalis and reads were partitioned among the clusters. Finally, Butterfly was used to process the individual graphs and ultimately report the full-length transcripts. To remove redundant sequences, CD-HIT was used to cluster the assembled highly similar transcripts into Unigenes [22], which can be accessed in the NCBI TAS platform under the accession number GIF00000000. BUSCO v4 was used to evaluate the completeness of the assembled Unigenes [23] using the eukaryota_odb10 dataset.

### 2.4. Annotation for the Transcriptome

We annotated the assembled transcriptome by aligning them to different databases. Initially, the transcriptome was searched against the NR (Non-Redundant Protein Sequence Database), NT (Nucleotide Sequence Database), KEGG (Kyoto Encyclopedia of Genes and Genomes) pathway, gene ontology (GO), Pfam, and UniProt/SwissProt databases using BLAST software, and hits with an e-value > 1 × 10^−5^ were filtered. Then, BLAST2GO was used to retrieve the GO annotation in terms of the biological process, cellular component, and molecular function [24]. Using the enzyme commission numbers produced by BLAST2GO, we mapped the assembled transcriptome to the KEGG pathway database and obtained the pathway annotation. rRNA transcripts were predicted using RNAMMER [25].

### 2.5. Likely Protein Identification and Annotation

We next extracted the likely proteins from the assembled transcriptome using TransDecoder. Then, the likely proteins were searched against the UniProtKB/Swiss-Prot database to identify known proteins, functional PFAM domains were identified using HMMER [26], signal peptides were predicted using SignalP [27], and transmembrane domains were predicted using TMHMM Sever v2.0 [28]. The EggNOG database v4.1 [29] was searched against to identify proteins in EuKaryotic Orthologous Groups (KOG), Clusters of Orthologous Groups (COGs), and non-supervised orthologous groups (NOGs). All the annotation for the assembled genes and likely proteins were subjected to the Trinotate v3.1.1 (http://trinotate.github.io) for combination.

Based on the gene annotation and likely protein annotation, we obtained the Unigenes, which were annotated into olfactory gene families, such as OBP, OR, IR, GR, and SNMP, using their names as key words. For the CSP transcripts, we aligned the Unigenes to all the CSP transcripts from NCBI GenBank. Hits with an e-value > 1 × 10^−5^ were filtered.

### 2.6. Noncoding Genes and microRNA Genes

Unannotated Ashmead genes were processed by the Coding Potential Calculator (CPC v2) with default parameters to identify potential long noncoding genes [30]. To identify potential microRNA (miRNA) genes, we first mapped all the animal mature miRNAs to the noncoding genes with a maximal of two mismatches [31]. Then, MIREAP was used to predict the miRNA precursors and MIRANDA was used to predict the target genes of these miRNAs [32].

### 2.7. Differential Expression Analysis

We aligned the clean reads of each sample to the Unigenes using Bowtie2 and profiled the gene expression using RSEM [33]. The trimmed mean of the M-values (TMM) method was used for normalization and edgeR was used to identify differentially expressed genes with the following cut-offs [34]: Count > 5, log_2_ fold change (log_2_ fc) >1 or log_2_ fc < −1, *p*-value < 0.05, and false discovery rate (FDR) < 0.05.

### 2.8. Function Enrichment Analysis

We calculated the *p*-value (calculated by Fisher’s exact test) and *q*-value (calculated by the R package ‘qvalue’) for each GO term and KEGG pathway involved in the differentially expressed genes. Enriched terms should satisfy the following criteria: *p*-value < 0.05 and *q*-value < 0.05.

### 2.9. Phylogenetic Analysis

Phylogenetic trees were reconstructed for OBPs and CSPs using MEGA7 software [35]. We obtained the likely protein sequences for the top 5 highly expressed OBP and CSP transcripts. These sequences together with the homology protein sequences, obtained from NCBI, were subjected to MEGA7 to create phylogenetic trees using the neighbor-joining method. The bootstrap procedure based on 1000 replicates was used to assess node support, and the node support values < 50% were not shown. Figtree v1.4.3 (https://github.com/rambaut/figtree/) was used to visualize the results.

### 2.10. qRT-PCR Verification

We used real-time quantitative reverse transcription polymerase chain reaction (qRT-PCR) to validate the expression levels of three randomly selected transcripts (TRINITY_DN1020_c0_g1_i3, TRINITY_DN1284_c0_g1_i11, and TRINITY_DN500_c0_g1_i2). Forward and reverse primers of the three transcripts and the internal control (β-actin) were predicted using Prime3 and synthesized at BGI-Shenzhen. The procedure of the qRT-PCR experiment was the same as the previous study [21]. Each transcript was measured three times in every sample and three independent repeats were performed (*n* = 9). The Delta cycle threshold (ΔCt) was used to present the expression of a transcript in the sample and ΔΔCt was used to show the expression difference between two samples. We used the relative normalized expression (RNE) to show the expression changes: $RNE=2^{-\mathrm{Ct}}$.

## 3. Results

### 3.1. Animal and Transcriptome Sequencing

**Figure 1:**
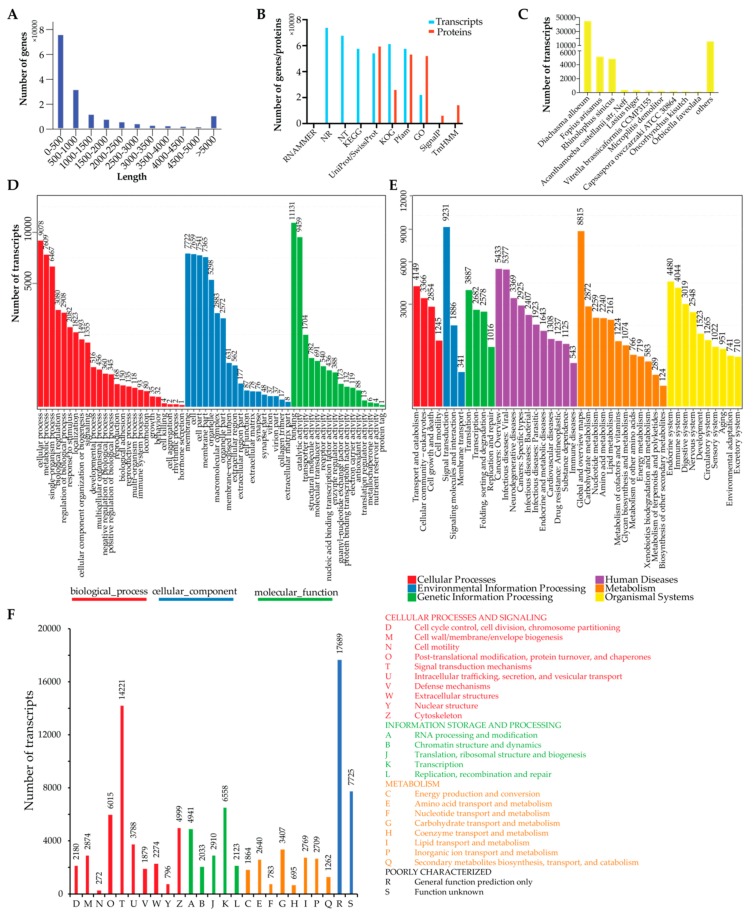
Overview of the assembled Ashmead transcriptome and annotation. (**A**) Length distribution of the assembled Ashmead transcriptome. (**B**) Number of transcripts and likely proteins aligned to different databases. (**C**) Number of the transcripts aligned to other species in the NR mapping results. (**D**) GO annotation for the assembled Ashmead transcriptome. (**E**) KEGG pathway annotation for the assembled Ashmead transcriptome. (**F**) KOG annotation for the assembled transcriptome.

After the Ashmead animals (three females and one male) were maintained with oriental fruit flies, which were fed guava (G), mango (M), and carambola ©, the antenna, head, breast, abdomen, and feet tissues were mixed together for RNA extraction and transcriptome sequencing. After data cleaning, we obtained a total of ~622.32 million reads (~69.15 million reads on average) and assembled 24,201 to 34,302 genes using Trinity for all the samples (Table 1). After similar genes/transcripts were clustered and merged, we finally obtained 162,621 Unigenes for the Ashmead transcriptome, with an average length of 1425.14 bp. The N50, GC content, and size of the transcriptome were calculated as 3572, 41.88%, and ~231 M, respectively (Table 1). Length distribution analysis showed that 53.05% of the total transcripts were longer than 500 bp and 11,101 transcripts (6.83% of the total transcripts) were longer than 5000 bp (Figure 1A). Last, we used BUSCO to evaluate the completeness of the assembled Unigenes and the results showed 99.6% of the assembled Unigenes were complete. In detail, out of the 255 evaluated BUSCOs in the dataset, 254 were complete, including 228 duplicated and 26 single-copy BUSCOs.

### 3.2. Transcriptome Annotation

We first annotated the assembled Ashmead transcriptome on the transcript level. All the transcripts were aligned to public databases for full annotation and Figure 1B showed 74,264, 68,185, 54,536, 58,109, 61,794, 58,113, and 22,546 transcripts were aligned to the NR, NT, UniProt/SwissProt, KEGG pathway, KOG, Pfam, and GO databases, respectively. Tthee NR mapping results (Figure 1C) showed the top 10 species aligned by the assembled Ashmead transcripts and the majority of the transcripts were aligned to *Diachasma alloeum* (45,569 transcripts), *Fopius arisanus* (5217 transcripts), and *Rhinolophus sinicus* (4920 transcripts). Unsurprisingly, the top two species together with Ashmead were all from the Braconidae family. GO annotation revealed that 11,131, 7722, and 9078 transcripts were involved in “binding”, “membrane”, and “cellular process”, respectively (Figure 1C). Then, we categorized the KEGG pathway annotation (Figure 1E) into six groups: Cellular progresses, environmental information processing, genetic information processing, human diseases, metabolism, and organism systems. Among them, “signal transduction” is the most significant pathway, which involved 9231 transcripts. KOG annotation also revealed that 14,221 transcripts were involved in the signal transduction mechanisms (Figure 1F).

Next, TranDecoder predicted 88,215 likely proteins encoded by the assembled Ashmead transcriptome and we annotated them using the Trinotate pipeline. It was shown that 59,887, 26,402, 53,725, and 52,572 were aligned to the UniProt/SwissProt, KOG, Pfam, and GO databases, respectively (Figure 1B). In addition, 6642 and 14,672 likely proteins were predicted to contain signal peptides and transmembrane helices (Figure 1B).

In addition, we predicted 74,963 of the unannotated transcripts using CPC2 had low coding probability. Interestingly, 2128 of the unannotated transcripts were predicted to be possible coding transcripts, which might be specific to the Ashmead and require further experiments to be verified. Next, we predicted 87 transcripts have the potential of producing miRNAs (Table S1). Notably, 84 of them were probably derived from the intron regions of coding genes while 1 and 3 derived from the Ashmead specific coding gene and long noncoding genes, respectively.

### 3.3. Olfactory Genes

We next identified genes encoding the olfactory gene from five families, including OBP, CSP, OR, GR, IR, and SNMP. In the Ashmead transcriptome, we found 43 transcripts encoding OBPs (Table 2, Table S2) by similarity and 35 of them were predicted to have complete ORFs by TransDecoder. The Ashmead OBPs were categorized into four sub-families: OBP-56, -69, -72, and -83 (Table S2). Multiple sequence alignment (Figure S1) showed these OBPs have 6 conserved cysteine residues (Cys) and SignalP predicted that 26 of these OBPs have the signal peptides located in the first 23 amino acids (aa) and 2 located in the first 37 aa. We identified that 69 transcripts had the potential of encoding CSPs in the Ashmead transcriptome by aligning the likely proteins to the known CSPs (Table 2, Table S2) and 63 of them had intact ORFs. SignalP identified 58 CSPs with the signal peptides in the first 28 aa while transmembrane domains were found in 13 CSPs. Surprisingly, 689 Ashmead transcripts had the capacity of encoding ORs and 115 transcripts were found to encode OR-13 (Table 2, Table S2). We also identified nine transcripts encoding OR coreceptors (OR-co). Out of the 347 OR transcripts that had intact ORFs, 276 were predicted to have transmembrane domains (Table 2). In the Ashmead transcriptome, there were 26 transcripts encoding IRs, including 3 IR-21, 18 IR-25, 4 IR-68, and 2 IR-93 (Table 2). Two thirds of the IR transcripts that had intact ORFs were predicted to have transmembrane domains. In addition, we identified 60 GR and 14 SNMP transcripts (Table 2). GR-28 and SNMP-1 were the largest group, which corresponded 19 and 11 transcripts, respectively. Further, we found 55 transcripts that had either the 7tm chemosensory receptor (PF02949) or GOBP (PF01395) PFAM domain in their protein sequences (Table 2, Table S2).

### 3.4. Gene Expression Profile

**Figure 2:**
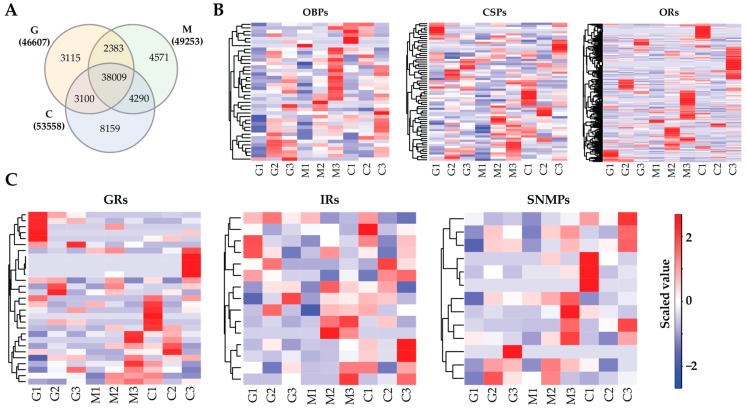
Expression levels of olfactory transcripts in the Ashmead insects. (**A**) Number of transcripts identified in the insects stimulated by three fruits. (**B**) Heat maps of the expression levels of OBP, CSP, and OR transcripts. (**C**) Heat maps showing the expression levels of transcripts encoding GRs, IRs, and SNMPs in the Ashmead insects.

We next profiled the gene expression in the Ashmead insects maintained with the fruit flies fed with the three kinds of fruits. After lowly expressed genes (count < 5) were filtered, RSEM identified a total of 63,627 transcripts in the Ashmead animals, of which 46,607, 49,253, and 53,558 transcripts were distributed in G, M, and C, respectively (Table S3). The Venn diagram (Figure 2A) revealed 38,009 transcripts commonly expressed in all samples while 3115, 4571, and 8159 were specifically detected in G, M, and C, respectively. Interestingly, not all the olfactory genes were expressed in the insects and we found 39 OBPs, 65 CSPs, 29 GRs, 382 ORs, 15 IRs, and 13 SNMPs. Figure 2B,C showed the expression levels of these olfactory transcripts in the insects, and revealed that different olfactory genes of Ashmead insects are responsible for the fruit flies with different fruits. According to the average expression levels, we showed the top five highly expressed olfactory transcripts identified in this study (Table 3) and it was revealed that the identities of highly expressed olfactory transcripts were shared by the parasitoid wasps of the fruit flies fed with different fruits. Notably, OBP56 and OBP69 were highly expressed in the insects; IR25a and SNMP1 were the only highly expressed transcript for the IR and SNMP groups, respectively.

### 3.5. Phylogenetic Analysis

**Figure 3:**
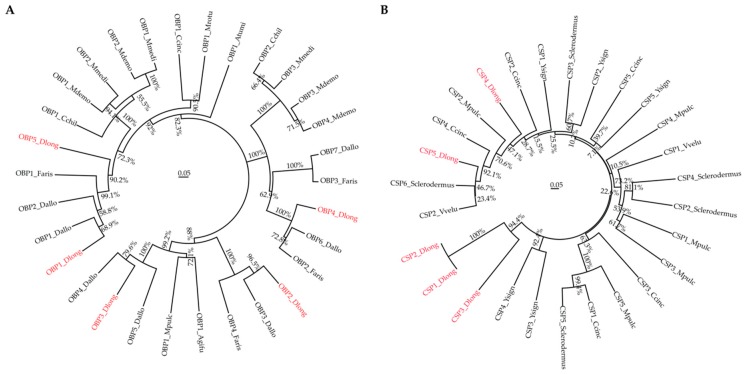
Phylogenetic trees for OBPs (**A**) and CSPs (**B**). Ashmead proteins are highlighted in red, the percentage represents the bootstrap value, and the scale bar represents the evolutionary distance.

We next compared the sequences of olfactory proteins identified in Ashmead and some other homology organisms and analyzed their phylogenetic relationship. First, we constructed the phylogenetic tree for the top 5 OBPs (Table 3) and 28 other OBP sequences obtained from NCBI. Detailed information, including accession numbers and species, can be accessed in Table S3. The phylogenetic tree of OBPs (Figure 3A) showed high similarity (68.9% to 96.5%) between Ashmead OBPs and other species, such as *Aethina tumida* (Atumi), *Aphidius gifuensis* (Agifu), *Cephus cinctus* (Ccinc), *Cotesia chilonis* (Cchil). *Diachasma alloeum* (Dallo), *Fopius arisanus* (Faris), *Megachile rotundata* (Mrotu), *Meteorus pulchricornis* (Mpulc), *Microplitis demolitor* (Mdemo), and *Microplitis mediator* (Mmedi). We next performed the phylogenetic analysis for CSPs. The top 5 highly expressed CSPs (Table 3) were compared with 22 CSPs from other species, like *Vespa velutina* (Vvelu), Ccinc, *Sclerodermus*, Mpulc, and *Yemma signatus* (Ysign). Detailed accession numbers of these CSPs can be accessed in Table S3. It is clear that the Ashmead CSPs can be clustered with other known CSPs. The phylogenetic analysis supported the identification and characterization of OBP and CSP transcripts in this study.

### 3.6. Differential Expression Analysis

**Figure 4:**
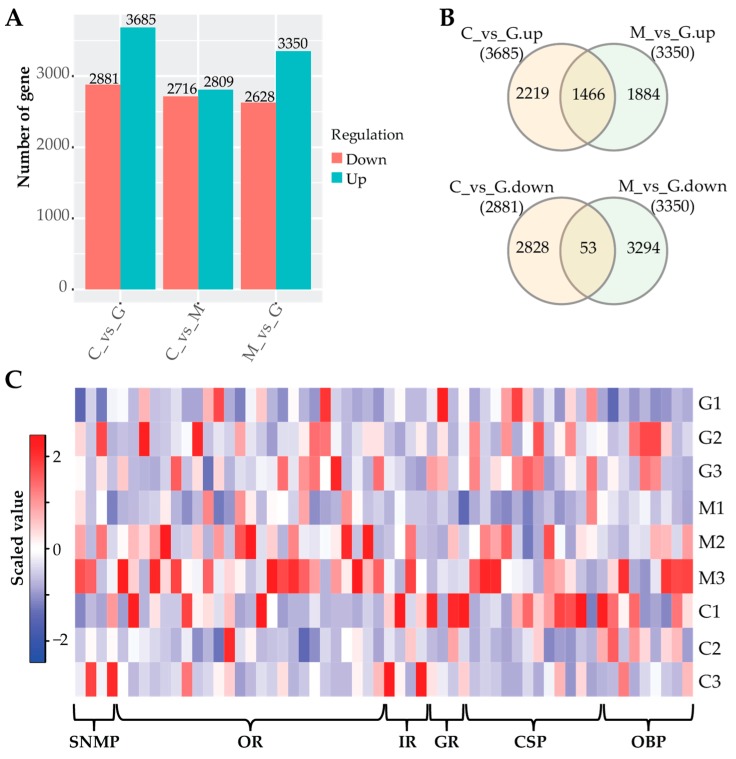
Differentially expressed transcripts in the parasitoid wasps of fruit flies fed with different fruits. (**A**) Number of differentially expressed transcripts in the insects in response to two fruit scents. (**B**) Venn diagram of up- (upper panel) and downregulated (lower panel) transcripts identified in C and M, compared to G. (**C**) A heat map showed the differential expression of 9 OBPs, 13 CSPs, 3 GRs, 4IRs, 25 ORs, and 4 SNMPs in the parasitoid wasps of fruit flies fed with different fruits.

Another important goal of this study was to identify genes in the insects in response to their parasitic hosts, which had difference fruit scents. Using edgeR, we identified a total of 2650 transcripts differentially expressed in the Ashmead insects in response to the fruit flies with different scents (Table S4), and the number of differentially expressed transcripts can be seen in Figure 4A. Compared to G, there were 1466 upregulated and 53 downregulated transcripts identified in both C and M (Figure 4B). Some transcripts were specifically expressed in the insects when they were parasitic to the fruit flies with one fruit scent (Figure 4B). We next analyzed the differentially expressed transcripts encoding olfactory proteins in the Ashmead insects in response to the three fruit flies. A total of 58 transcripts encoding olfactory proteins were found, including 9 OBPs, 13 CSPs, 3 GRs, 4IRs, 25 ORs, and 4 SNMPs (Figure 4C). This evidence further supports the existence of multiple pathways of Ashmead insects in response to different fruits scents. In addition to olfactory proteins, some other protein families were differentially expressed in the wasps maintained with fruit flies supplied with different fruits, such as 2 LOC107047718 (Putative 7 transmembrane sweet-taste receptor of 3 gcpr), 88 ribosomal proteins, 109 transcription factors, 99 histones, 11 heat shock proteins, and 43 G-protein coupled receptors/regulators (Table S4). The differential expression of transcripts from different families indicated the complicated regulation mechanisms of parasitoid wasps in response to their hosts with different fruit scents. More experiments are required to explore their functions in this process.

### 3.7. qRT-PCR

We further used qRT-PCR to validate the expression levels of three randomly selected transcripts in the parasitoid wasps. The primers for the three transcripts and internal control (actin) were predicted using Prime3 and can be accessed in Table 4. We used Log2FC and RNE to show the expression changes of the transcripts in C, M, and G identified by RNA-Seq and qRT-PCR, respectively. Overall, the expression patterns of these transcripts in most comparisons were consistent by both RNA-Seq and qRT-PCR except three (TRINITY_DN1020_c0_g1_i3, TRINITY_DN500_c0_g1_i2 in C_vs_M, and TRINITY_DN1020_c0_g1_i3 in M_vs_G). The high agreement of the gene expression patterns in RNA-Seq and qRT-PCR indicates that the transcripts identified in this study might be functionally expressed in the parasitoid wasps maintained with fruit flies with different scents, which requires future functional experiments.

## 4. Discussion

This project was initiated in an effort to identify the olfactory proteins of parasitoid wasps. In recent years, many aspects about the perception of pheromones and other odorants have been elucidated [36]. Olfaction is used by insects to recognize volatile cues that allow the detection of food, predators, and mates [37]. We identified 43 OBPs, 69 CSPs, 60 GRs, 689 ORs, 26 IRs, and 14 SNMPs in *D. longicaudata* (Table 2, Table S2) and some of them were differentially expressed when they were maintained with fruit flies fed with different fruits (Figure 4C, Table S4). This is the first time the chemosensory genes in *D. longicaudata* have been investigated and our findings provide a basis for elucidating the molecular mechanisms of the olfactory-related behaviors of parasitoid wasps.

OBPs are a group of proteins that specialize in the transport of lipids. In this study, we identified 43 transcripts encoding OBPs and this number is similar to the number of genes encoding OBPs in the *Drosophila* genome [38]. Addittionally, classic OBPs have been reported to contain six Cys in their sequences [38,39]. Because all the identified OBPs in this study were general OBPs (GOBPs) (Table S2), we found conserved Cys in their sequences (Figure S1), which increased the confidence of using the transcriptome to identify the OBPs in *D. longicaudata*. In detail, the *D. longicaudata* OBPs were grouped into four subfamilies: OBP-56, -69, -72, and -83 (Table S2). However, no studies have been demonstrated to distinguish their functions. In general, according to their names, they are called GOBPs because they bind general odorants that are likely to be represented by the same volatiles for most of the species [40]. We also showed the differential expression of nine transcripts encoding OBPs in parasitoid wasps in response to the fruit flies with different scents (Figure 4, Table S4). This might indicate that some specific OBPs are expressed to discriminate different scents.

Similar to OBPs, CSPs are another group of proteins that mediate the olfactory recognition in insects [41]. They are thought to be expressed during nearly the whole life circle of insects [42,43]. The number of CSP genes varies in species. For example, only 4~8 CSP genes in ants, flies, bees, wasps, and anopheline mosquitoes [44]; 19~20 in butterfly, moth, and beetle [45,46,47]; and 27 to 83 in *Culex* mosquito species [48]. We identified 69 CSP transcripts produced by 58 genes (Table S2), the number of which is similar to *Culex* mosquito species. CSPs function not only in the chemical communication between insects and the environment but also in some other cellular processes, such as lipid transport, general immunity, insecticide resistance, and xenobiotic degradation [45,49]. In ants, CSPs have been proposed to mediate the recognition of chemical signatures composed of cuticular lipids [50]. The differential expression of CSP transcripts identified in this study (Table S4) may support the ability to recognize different scents from their hosts.

In the present study, we also identified some other olfactory gene families, such as OR, IR, GR, and SNMP, which were differentially expressed in response to the fruit fly with different scents (Table S2, Table S4). OR is the name for all molecules that are expressed in the cell membranes of olfactory receptor neurons and are responsible for the detection of odorants. The ORs form a multigene family consisting of around 800 genes in humans and 1400 genes in mice [51]. We identified 689 OR transcripts derived from 637 genes (Table 2, Table S2). The diversity of ORs might help insects to discriminate as many different odors as possible. GRs are found be expressed exclusively in gustatory receptor neurons [52]. However, many GRs are not related to taste receptors but function in the detection of sugars, bitter compounds, and non-volatile pheromones [53]. Interestingly, GR28B represents a new class of thermosensor and is required for thermotaxis [54]. We found GR28B differentially expressed in C and M (Figure 4C, Table S2). This might be evidence of its new role in the detection of different scents. IR genes are expressed in coeloconic sensilla of the antenna and respond, among others, to water and amines [55]. IRs are not related to insect ORs but rather have evolved from ionotropic glutamate receptors (iGluRs), a conserved family of synaptic ligand-gated ion channels [56]. In this study, we identified 26 IR transcripts, and 18 of them encode IR25A (Table 2, Table S2). It is conceivable that the IR25A ancestor initially evolved as a sensory detector for external glutamate, analogous to the synaptic function of iGluRs, and that it only later acquired a co-receptor function after duplication and diversification of the IR repertoire [56]. We also identified 14 SNMP transcripts in the parasitoid wasps, including 11 encoding SNMP1 and 3 encoding SNMP2 (Table 2, Table S2). The SNMP1 has been shown to be antenna specific and play an important role in pheromone detection [57]. While SNMP2, which acts as a second lepidoperan and also associates with pheromone-sensitive sensilla, has been shown to be expressed in sensilla support cells rather than neurons [58,59]. The identification and differential expression of olfactory-related transcripts revealed the complex chemosensory system of *D. longicaudata* and supported a diverse function of olfactory genes in discriminating different chemical cues.

The limitations of this project may include the use of the tissue mixture of insects. It is said that some OBPs are expressed in insects with a sex preference. For example, MsepOBP5 exhibited female-biased expression in 0- and 5-day-old adults; MsepOBP22 displayed female-biased expression in 0- and 5-day-old adults but was male-biased in 3-day-old adults [60]. Due to the difficulty of the sample preparation, it is hard to get enough material for sequencing with the same sex. Additionally, it is difficult to determine the tissue-specific olfactory genes. However, our findings provide a basis of future studies about the olfactory system in *D. longicaudata*.

## 5. Conclusions

In conclusion, we assembled the first transcriptome for *D. longicaudata* using transcriptome sequencing and identified 43 OBPs, 69 CSPs, 60 GRs, 689 ORs, 26 IRs, and 14 SNMPs. Further, 9 OBPs, 13 CSPs, 3 GRs, 4IRs, 25 ORs, and 4 SNMPs were differentially expressed in the insects in response to fruit flies with different scents. Our findings provide a basis towards understanding the molecular mechanisms of *D. longicaudata* in the detection of chemosensory cues. Additionally, the sequences including olfactory genes, noncoding genes, and miRNAs identified in this study can be used in the future and benefit other researchers in this field.

## Figures and Tables

**Table 1:** Overview of the de novo transcriptome of Ashmead.

	G1	G2	G3	M1	M2	M3	C1	C2	C3
Clean reads	67,492,576	74,983,476	70,212,518	78,796,858	61,848,876	72,961,796	71,005,592	54,703,286	70,316,132
Assembled genes	34,302	26,032	25,398	24,201	33,375	33,081	33,308	24,772	24,707
Assembled transcripts	49,914	40,912	39,528	38,089	50,517	51,704	50,530	38,856	39,637
Unigenes	162,621								
Mean Length (bp)	1425.14								
N50	3572								
GC (%)	41.88								
Total bases	231,757,796								
Expressed transcripts	10,736	10,320	10,645	9860	10,863	11,695	16,827	11,765	14,728

**Table 2:** Olfactory genes identified in the Ashmead transcriptome.

Type	Transcripts	Sub-Family	Intact ORF	SignalP	TMHMM
OBP	43	21 OBP-56, 7 OBP-69, 4 OBP-72, 11 OBP-83	35	28	15
CSP	69	5 CSP-1, 3 CSP-3, 2 CSP-4, 5 CSP-5, 10 CSP-6, 3 CSP-7, 4 CSP-8, 37 CSP *	63	50	13
OR	689	45 OR-1, 6 OR-1F12, 21 OR-10, 115 OR-13, 42 OR-2, 46 OR-22, 2 OR-23, 9 OR-24, 2 OR-245, 1 OR-260, 2 OR-266, 1 OR-277, 15 OR-30, 6 OR-33, 31 OR-4, 5 OR-42, 14 OR-43, 2 OR-45, 24 OR-46, 47 OR-47, 26 OR-49, 2 OR-59, 1 OR-5, 1 OR-63, 48 OR-67, 1 OR-69, 6 OR-71, 13 OR-7, 1 OR-81, 62 OR-82, 37 OR-85, 11 OR-92, 11 OR-94, 5 OR-98, 4 OR-9, 9 OR-co, 1 OR-142, 14 OR *	347	25	276
IR	26	3 IR-21, 18 IR-25, 4 IR-68, 2 IR-93	18	4	12
GR	60	6 GR-2, 5 GR-107, 3 GR-15, 1 GR-23, 19 GR-28, 4 GR-2, 8 GR-43, 2 GR-64, 3 GR-66, 8 GR *	26	1	25
SNMP	14	11 SNMP-1, 3 SNMP-3	10	1	9
Others ^a^	54	16 CR, 38 GOBP	10	12	22

^a^ Transcripts containing the chemosensory receptor or the GOBP domain, by PFAM annotation. * Products are not specified.

**Table 3:** Top five transcripts encoding olfactory proteins in Ashmead insects.

TranscriptID	G ^a^	M ^a^	C ^a^	Protein/Gene	Description
**OBP**					
TRINITY_DN22_c0_g3_i1	41,518.89	55,018.99	39,228.41	OB56D	General odorant-binding protein 56d
cluster_contig4670	20,459.11	27,957.59	83,228.40	OB56H	General odorant-binding protein 56h
cluster_contig3738	15,552.04	13,752.58	20,997.13	OB69A	General odorant-binding protein 69a
TRINITY_DN3641_c0_g2_i1	5,010.50	13,074.19	4,838.31	OB69A	General odorant-binding protein 69a
TRINITY_DN23528_c0_g1_i1	3853.93	5367.95	6228.73	OB56D	General odorant-binding protein 56d
**CSP**					
TRINITY_DN1018_c0_g1_i5	11,065.74	15,620.31	10,030.62	CSP	chemosensory protein
TRINITY_DN661_c1_g1_i1	9447.67	10,194.33	7724.67	THK33221.1	chemosensory protein 4
TRINITY_DN4258_c0_g1_i1	4538.67	4738.33	5976.67	AZQ24964.1	chemosensory protein, partial
TRINITY_DN1848_c0_g1_i6	5121.01	3247.27	4919.50	CSP	chemosensory protein
cluster_contig14748	3679.27	3944.22	2395.00	THK33222.1	chemosensory protein 5
**OR**					
cluster_contig6330	41,364.85	68,243.05	24,104.78	LOC107043577	Odorant receptor
TRINITY_DN1357_c0_g1_i5	18,042.97	23,565.93	11,831.46	OR43A	Odorant receptor 43a
TRINITY_DN1357_c0_g1_i6	7765.79	27,596.19	13,421.25	OR43A	Odorant receptor 43a
cluster_contig12468	16,698.80	23,528.66	8172.54	LOC107043576	Odorant receptor
cluster_contig9759	2835.24	2248.79	3479.03	OR43A	Odorant receptor 43a
**GR**					
TRINITY_DN9119_c0_g1_i2	455.68	489.30	545.92	GR107	gustatory receptor Gr107
TRINITY_DN3525_c0_g1_i10	288.94	333.98	230.17	GR43A	gustatory receptor for sugar taste 43a-like
cluster_contig36	290.10	43.82	30.95	GR43a	gustatory receptor for sugar taste 43a-like
TRINITY_DN14061_c0_g1_i3	91.79	116.05	78.74	GR43a	gustatory receptor for sugar taste 43a-like
TRINITY_DN4338_c0_g1_i4	90.22	77.22	64.01	GR107	gustatory receptor Gr107
**IR**					
TRINITY_DN11328_c0_g1_i1	41.09	39.27	45.12	IR25A	Ionotropic receptor 25a
cluster_contig8505	39.77	34.65	39.58	IR25A	Ionotropic receptor 25a
TRINITY_DN4424_c0_g1_i2	16.26	12.92	47.84	IR25A	Ionotropic receptor 25a
cluster_contig11752	19.21	25.57	18.80	IR25A	Ionotropic receptor 25a
TRINITY_DN13912_c1_g2_i1	20.88	10.71	20.37	IR25A	Ionotropic receptor 25a
**SNMP**					
TRINITY_DN1934_c0_g1_i1	3567.61	3632.31	5117.35	SNMP1	Sensory neuron membrane protein 1
TRINITY_DN4669_c1_g1_i1	176.96	204.92	206.30	SNMP1	Sensory neuron membrane protein 1
TRINITY_DN1867_c0_g2_i1	86.72	83.98	50.41	XP_015114627.1	Sensory neuron membrane protein 1-like
cluster_contig4264	70.20	73.23	46.97	SNMP1	Sensory neuron membrane protein 1
cluster_contig237	44.54	90.10	34.07	SNMP1	Sensory neuron membrane protein 1

^a^ Normalized expression, TMM.

**Table 4:** qRT-PCR validation. Log_2_ FC represents the log_2_ values of the fold change of a transcript identified by the RNA-Seq while RNE represents the relative normalized expression of a transcript identified by qRT-PCR.

		TRINITY_DN1020_c0_g1_i3	TRINITY_DN1284_c0_g1_i11	TRINITY_DN500_c0_g1_i2
Primers	Forward	CAACTTCAAGAACAATCCGACAAC	ACTTATAGACGCATGCCAAGACC	GACGTCGCTATGAACGCTTG
	Reverse	CCACAGCCAGAGACACAGC	GGGCTGGAGAACGGGGATG	GATTCTGATTTCCAGTACGAATACG
C_vs_G	Log_2_ FC	10.00	9.84	0
	p-value	4.12 × 10^−28^	6.02 × 10^−27^	1
	RNE	1.13	2.32	0.42
C_vs_M	Log_2_ FC	−9.47	1.43	6.43
	p-value	2.28 × 10^−23^	0.0013	1.98 × 10^−33^
	RNE	1.68	0.66	0.00
M_vs_G	Log_2_ FC	−0.53	8.32	6.66
	p-value	0.3856	1.13 × 10^−15^	7.18 × 10^−34^
	RNE	0.67	3.52	79.36

## Supplementary Materials

The following are available online at https://www.mdpi.com/2073-4425/11/2/144/s1, Figure S1: Multiple sequence alignment of OBP sequences identified in this study. It showed the conserved six Cys (C1~C6) in these sequences (highlighted in yellow and marked with *). Table S1: miRNA precursors identified in the parasitoid wasp transcriptome. Table S2: Expression profile of transcripts encoding olfactory proteins. Table S3: OBP and CSP sequences for the phylogenetic analysis. Table S4: Differentially expressed transcripts in the parasitoid wasps of fruit flies fed with different fruits.
