# Supplementary material for: De Novo Transcriptome Identifies Olfactory Genes in Diachasmimorpha longicaudata (Ashmead)
Source: Genes (Basel). 2020 Jan 29;11(2):144. doi: 10.3390/genes11020144 (PMC7074194; doi:10.3390/genes11020144)
Supplement: Supplementary file 1 [file genes-11-00144-s001.gz › Figure S1.pdf]

```
                                                                                                                C1                          C2  C3                                         C4           C5       C6
                                                                                                                *                           *   *                                          *            *        *
TRINITY_DN17498_c0_g1_i1  --------------------MKF-------------------------------LVVVIFVCLAGAL-AQELTEAQRRRLREHRDICIRETDANRAEVDRARQ-GQWADNPQIRCFALCMMRRLRLMSEDGQLNEAAARQRLALVVPR----ERVEEIMTKC-KDLKGNTP---CDTGYLVLKCYTDNRANVVX----------
TRINITY_DN1796_c0_g1_i1   --------------MARHMVCCFL------------------------------IGMAMQALLVSAGRPDFITDDMMAMVADDKARCMSEHGTTEALIDEVNT-GALPNNRALTCYMDCLFAAFGVI-DEGELEVDMLVGFLPDHMQD-----AARDLLETC-AKQPGADP---CDKVFEIAKCVQAKRPDLWFMIX-------
TRINITY_DN1838_c0_g1_i1   -------------------MVKYI------------------------------VSAFIAVCLVAAIRAGEIPPEFKEIAPEVRRVCLEESGAENEWVVKANK-GDFTDDPKFKCYLKCTLAQFGAVSRKG-VNFDALTKLAP--PAY---KEILDKVISAC-KDTKPTIPGDVCDQVYEASKCFYRAAPDNYFVMX-------
TRINITY_DN1916_c0_g1_i1   --------------MARHMVCCFL------------------------------IGMAMQALLVSAGRPDFITDDMMAMVADDKARCMSEHGTTEALIDEVNT-GALPNNRALTCYMDCLFAAFGVI-DEGELEVDMLVGFLPDHMQD-----AARDLLETC-AKQPGADP---CDKVFEIAKCVQAKRPDLWFMIX-------
TRINITY_DN2097_c0_g1_i2   --------------------MKV-------------------------------FVVILSTCLIGAL-AQELTEAQKQKVRENRDACITETGADREEVNKANK-GEWADNAKIRCFTLCMLKKWGMMNDAGVLDEAAARQKMGLQMKP----EKVEEIMTKC-KYLKGDTA---CDTAYMMMKCYTDNRAVTVX----------
TRINITY_DN2097_c0_g1_i2   --------------------MKF-------------------------------LVVVIFVCLAGAL-AQELTEAQRRRLREHRDICIRETDANRAEVDRARQ-GQWADNPQIRCFALCMMRRLRLMSEDGQLNEAAARQRLALVVPR----ERVEEIMTKC-KDLKGNTP---CDTGYLVLKCYTDNRANVVX----------
TRINITY_DN2097_c0_g1_i2   MRLAMKKSSHCVRLVPDAYIGGHFLQTIFTYQHAFATGSNFRELRIIQQLIMKFLVIAVLVCIVGAL-ASEQRETPEARRQRYRDACRAETGADQADIDKARK-GEWADKENLRCYTLCMLKKGNLMNDEGVLDEIVTRDTLARKLPA----EQINDIMAKC-KDLKGSNA---CGTAQRIMKCYNEQKTHLMX----------
TRINITY_DN22_c0_g3_i1     --------------------MKV-------------------------------FVVILSTCLIGAL-AQELTEAQKQKVRENRDACITETGADREEVNKANK-GEWADNAKIRCFTLCMLKKWGMMNDAGVLDEAAARQKMGLQMKP----EKVEEIMTKC-KYLKGDTA---CDTAYMMMKCYTDNRAVTVX----------
TRINITY_DN23528_c0_g1_i1  ------------MCLLQTVKMKLL------------------------------VILLIFHVAFA---AGAFRPADIVRFQRAVEKCRISEDVSDEVLERVLN-GEIVDDPHFNCFAACLLEEFQLLKPDGSFNDELAVTKIPQDADF---AQQLTDAIKTC-SARRDPDK---CTTAHLLFVCMYENNIPTLLFGX-------
TRINITY_DN2432_c0_g2_i1   --------WEVLVEFSDSIHQKFFFLDSQSKNQNSKMKSAFISGLV--------IVLVVLNFDNVG--AKMTIPQVTNMLMPMRKTCMQKTGASAELVDAPKT-GPMPDDPVLQCYYACLLKMIKVLTKEGLPNVDSMVKQMDVMLPADDITARLKEVIALCTPAVTSTDS---CEGTWQFIKCFYETDKNVCFFPX-------
TRINITY_DN25035_c0_g1_i1  MRLAMKKSSHCVRLVPDAYIGGHFLQTIFTYQHAFATGSNFRELRIIQQLIMKFLVIAVLVCIVGAL-ASEQRETPEARRQRYRDACRAETGADQADIDKARK-GEWADKENLRCYTLCMLKKGNLMNDEGVLDEIVTRDTLARKLPA----EQINDIMAKC-KDLKGSNA---CGTAQRIMKCYNEQKTHLMX----------
TRINITY_DN2526_c0_g2_i2   ---------MGIKALPIIQRENFK---------------RRSTGID--------MRIIGVSLALLSL-VLFVRGDDKDPHGPIREKCKDQFGLSSDDL----K-AAMEDPSDVGCYILCFFKDLSIMDDSGKFDPDAALDAIEDSAKD-----DAKPVLSSCDSKVKKSTTKDPCARALEVVTCFKEEAPELYKNLGIFHPLGX
TRINITY_DN26386_c0_g1_i1  --------------------MNTS------------------------------TVVLVFCALAVTF---VSGGDMKAEMHAQVEKCIIETGVDPSVLKSLHETGGANADENVKCLGACIMKGLGVMAADGTVNLDKAKSLVPSDAPD---HDALLAAVEEC-HAEKGAND---CETAHAIGMCMHKKHVGPMDX---------
TRINITY_DN26629_c0_g1_i1  NNYLARCFRRHIAQYSFSMKSGLF--------------------VV--------ALVGAFAVAAGGP-PDKDCPLMKA-LKESIDACIDK--LSEESVKLMEK-DAFADNEEIRCFHACVMTHSGLMTD-GKMDIPALEEMLSGNPDDEEEAQKIIEIMKMCKPEAEISDN--ECEVAGNYVKCFQAKKVNX------------
TRINITY_DN2842_c0_g2_i1   --------------------MKF-------------------------------LVVVIFVCLAGAL-AQELTEAQRRRLREHRDICIRETDANRAEVDRARQ-GQWADNPQIRCFALCMMRRLRLMSEDGQLNEAAARQRLALVVPR----ERVEEIMTKC-KDLKGNTP---CDTGYLVLKCYTDNRANVVX----------
TRINITY_DN2842_c0_g3_i1   MRLAMKKSSHCVRLVPDAYIGGHFLQTIFTYQHAFATGSNFRELRIIQQLIMKFLVIAVLVCIVGAL-ASEQRETPEARRQRYRDACRAETGADQADIDKARK-GEWADKENLRCYTLCMLKKGNLMNDEGVLDEIVTRDTLARKLPA----EQINDIMAKC-KDLKGSNA---CGTAQRIMKCYNEQKTHLMX----------
TRINITY_DN2842_c0_g3_i1   --------------------MKV-------------------------------FVVILSTCLIGAL-AQELTEAQKQKVRENRDACITETGADREEVNKANK-GEWADNAKIRCFTLCMLKKWGMMNDAGVLDEAAARQKMGLQMKP----EKVEEIMTKC-KYLKGDTA---CDTAYMMMKCYTDNRAVTVX----------
TRINITY_DN2902_c0_g1_i1   ---------MGIKALPIIQRENFK---------------RRSTGID--------MRIIGVSLALLSL-VLFVRGDDKDPHGPIREKCKDQFGLSSDDLKAAME-----DPSDVGCYILCFFKDLSIMDDSGKFDPDAALDAIEDSAKD-----DAKPVLSSCDSKVKKSTTKDPCARALEVVTCFKEEAPELYKNLGIFHPLGX
TRINITY_DN30107_c0_g1_i1  NNYLARCFRRHIAQYSFSMKSGLF--------------------VV--------ALVGAFAVAAGGP-PDKDCPLMKA-LKESIDACIDK--LSEESVKLMEK-DAFADNEEIRCFHACVMTHSGLMTD-GKMDIPALEEMLSGNPDDEEEAQKIIEIMKMCKPEAEISDN--ECEVAGNYVKCFQAKKVNX------------
TRINITY_DN30439_c0_g1_i4  --------------------MNTS------------------------------TVVLVFCALAVTF---VSGGDMKAEMHAQVEKCIIETGVDPSVLKSLHETGGANADENVKCLGACIMKGLGVMAADGTVNLDKAKSLVPSDAPD---HDALLAAVEEC-HAEKGAND---CETAHAIGMCMHKKHVGPMDX---------
TRINITY_DN3053_c0_g1_i2   --------GKYSWNSVIRYIRNFFFLDSQSKNQNSKMKSAFISGLV--------IVLVVLNFDNVG--AKMTIPQVTNMLMPMRKTCMQKTGASAELVDAPKT-GPMPDDPVLQCYYACLLKMIKVLTKEGLPNVDSMVKQMDVMLPADDITARLKEVIALCTPAVTSTDS---CEGTWQFIKCFYETDKNVCFFPX-------
TRINITY_DN3355_c0_g1_i1   ---------MGIKALPIIQRENFK---------------RRSTGID--------MRIIGVSLALLSL-VLFVRGDDKDPHGPIREKCKDQFGLSSDDLKAAME-----DPSDVGCYILCFFKDLSIMDDSGKFDPDAALDAIEDSAKD-----DAKPVLSSCDSKVKKSTTKDPCARALEVVTCFKEEAPELYKNLGIFHPLGX
TRINITY_DN3475_c0_g1_i4   ----------------MRKYVGFLC-------------------LI--------LQVSIGHCGPVGR-PDFVSDEMIALAASVVNACQTQTGVATADIEAVRN-GQWLNSTPLKCYMYCLWEQFGLVDDKRELSLNGMLTFFQRIPAY---RAEVQTAIRECKKIADGDN----CQYAYTFNLCYSKLSPRTYYLFX-------
TRINITY_DN3475_c0_g1_i4   ----------------MRKYVGFLC-------------------LI--------LQVSIGHCGPVGR-PDFVSDEMIALAASVVNACQTQTGVATADIEAVRN-GQWLNSTPLKCYMYCLWEQFGLVDDKRELSLNGMLTFFQRIPAY---RAEVQTAIRECKKIGKFFADGDNCQYAYTFNLCYSKLSPRTYYLFX-------
TRINITY_DN36216_c0_g1_i1  --------------------MNTS------------------------------TVVLVFCALAVTF---VSGGDMKAEMHAQVEKCIIETGVDPSVLKSLHETGGANADENVKCLGACIMKGLGVMAADGTVNLDKAKSLVPSDAPD---HDALLAAVEEC-HAEKGAND---CETAHAIGMCMHKKHVGPMDX---------
TRINITY_DN3638_c0_g1_i1   ---------EVLVEFSDSIHQKFFFLDSQSKNQNSKMKSAFISGLV--------IVLVVLNFDNVG--AKMTIPQVTNMLMPMRKTCMQKTGASAELVDAPKT-GPMPDDPVLQCYYACLLKMIKVLTKEGLPNVDSMVKQMDVMLPADDITARLKEVIALCTPAVTSTDS---CEGTWQFIKCFYETDKNVCFFPX-------
TRINITY_DN3641_c0_g2_i1   -------------------MVKYI------------------------------VSAFIAVCLVAAIRAGEIPPEFKEIAPEVRRVCLEESGAENEWVVKANK-GDFTDDPKFKCYLKCTLAQFGAVSRKG-VNFDALTKLAP--PAY---KEILDKVISAC-KDTKPTIPGDVCDQVYEASKCFYRAAPDNYFVMX-------
TRINITY_DN3807_c0_g1_i3   ----------------MRKYVGFLC-------------------LI--------LQVSIGHCGPVGR-PDFVSDEMIALAASVVNACQTQTGVATADIEAVRN-GQWLNSTPLKCYMYCLWEQFGLVDDKRELSLNGMLTFFQRIPAY---RAEVQTAIRECKKIGKFFADGDNCQYAYTFNLCYSKLSPRTYYLFX-------
TRINITY_DN4374_c0_g1_i4   ----------------MRKYVGFLC-------------------LI--------LQVSIGHCGPVGR-PDFVSDEMIALAASVVNACQTQTGVATADIEAVRN-GQWLNSTPLKCYMYCLWEQFGLVDDKRELSLNGMLTFFQRIPAY---RAEVQTAIRECKKIGKFFADGDNCQYAYTFNLCYSKLSPRTYYLFX-------
TRINITY_DN5058_c0_g1_i1   ----------------MRKYVGFLC-------------------LI--------LQVSIGHCGPVGR-PDFVSDEMIALAASVVNACQTQTGVATADIEAVRN-GQWLNSTPLKCYMYCLWEQFGLVDDKRELSLNGMLTFFQRIPAY---RAEVQTAIRECKKIGKFFADGDNCQYAYTFNLCYSKLSPRTYYLFX-------
TRINITY_DN5692_c0_g1_i2   NNYLARCFRRHIAQYSFSMKSGLF--------------------VV--------ALVGAFAVAAGGP-PDKDCPLMKA-LKESIDACIDK--LSEESVKLMEK-DAFADNEEIRCFHACVMTHSGLMTD-GKMDIPALEEMLSGNPDDEEEAQKIIEIMKMCKPEAEISDN--ECEVAGNYVKCFQAKKVNX------------
TRINITY_DN7227_c0_g2_i1   ----------------QTVKMKLL------------------------------VILLIFHVAFA---AGAFRPADIVRFQRAVEKCRISEDVSDEVLERVLN-GEIVDDPHFNCFAACLLEEFQLLKPDGSFNDELAVTKIPQDADF---AQQLTDAIKTC-SARRDPDK---CTTAHLLFVCMYENNIPTLLFGX-------
cluster_contig12763       ----------------MRKYVGFLC-------------------LI--------LQVSIGHCGPVGR-PDFVSDEMIALAASVVNACQTQTGVATADIEAVRN-GQWLNSTPLKCYMYCLWEQFGLVDDKRELSLNGMLTFFQRIPAY---RAEVQTAIRECKKIGKFFADGDNCQYAYTFNLCYSKLSPRTYYLFX-------
cluster_contig3353        ------------MCLLQTVKMKLL------------------------------VILLIFHVAFA---AGAFRPADIVRFQRAVEKCRISEDVSDEVLERVLN-GEIVDDPHFNCFAACLLEEFQLLKPDGSFNDELAVTKIPQDADF---AQQLTDAIKTC-SARRDPDK---CTTAHLLFVCMYENNIPTLLFGX-------
cluster_contig3738        -------------------MVKYI------------------------------VSAFIAVCLVAAIRAGEIPPEFKEIAPEVRRVCLEESGAENEWVVKANK-GDFTDDPKFKCYLKCTLAQFGAVSRKG-VNFDALTKLAP--PAY---KEILDKVISAC-KDTKPTIPGDVCDQVYEASKCFYRAAPDNYFVMX-------
cluster_contig4520        --------------------MKV-------------------------------FVVILSTCLIGAL-AQELTEAQKQKVRENRDACITETGADREEVNKANK-GEWADNAKIRCFTLCMLKKWGMMNDAGVLDEAAARQKMGLQMKP----EKVEEIMTKC-KYLKGDTA---CDTAYMMMKCYTDNRAVTVX----------
cluster_contig4670        MRLAMKKSSHCVRLVPDAYIGGHFLQTIFTYQHAFATGSNFRELRIIQQLIMKFLVIAVLVCIVGAL-ASEQRETPEARRQRYRDACRAETGADQADIDKARK-GEWADKENLRCYTLCMLKKGNLMNDEGVLDEIVTRDTLARKLPA----EQINDIMAKC-KDLKGSNA---CGTAQRIMKCYNEQKTHLMX----------
cluster_contig6435        -----FTYWEVLVEFSDSIHQKFFFLDSQSKNQNSKMKSAFISGLV--------IVLVVLNFDNVG--AKMTIPQVTNMLMPMRKTCMQKTGASAELVDAPKT-GPMPDDPVLQCYYACLLKMIKVLTKEGLPNVDSMVKQMDVMLPADDITARLKEVIALCTPAVTSTDS---CEGTWQFIKCFYETDKNVCFFPX-------
cluster_contig6516        --------------------MKF-------------------------------LVVVIFVCLAGAL-AQELTEAQRRRLREHRDICIRETDANRAEVDRARQ-GQWADNPQIRCFALCMMRRLRLMSEDGQLNEAAARQRLALVVPR----ERVEEIMTKC-KDLKGNTP---CDTGYLVLKCYTDNRANVVX----------
```
